# Right-sided infective endocarditis complicated with aortic pseudoaneurysms and systemic embolism in a 27-year-old female with ventricular septal defect: a case report

**DOI:** 10.11604/pamj.2024.48.174.33183

**Published:** 2024-08-13

**Authors:** Zineb Agoumy, Abdoul Wahab Karimou, Nawal Doghmi, Mohamed Cherti

**Affiliations:** 1 Department of Cardiology B, Hospital University Center Ibn Sina, Mohamed V University, Rabat, Morocco

**Keywords:** Infective endocarditis, ventricular septal defect, paradoxical embolism, pseudo-aneurysm, case report

## Abstract

Infective endocarditis (IE) is one of the most frequent complications of ventricular septal defect (VSD) in adults, but is rarely associated with multiple systemic embolisms and aortic pseudoaneurysms. The authors report a case of a 27-year-old female known to have an asymptomatic neglected VSD. She was admitted to our unit with complaints of prolonged fever and chills. Physical examination detected hyperthermia at 38.7°C and pathognomonic holosystolic murmur detected by cardiac auscultation. Laboratory blood test showed evidence of acute staphylococcus infection and imaging investigations revealed perimembranous restrictive VSD, with vegetations exclusively present in the right heart ventricle, in addition to mycotic aneurysms on the aortic arch and multiple systemic embolis. A targeted antibiotic therapy was initiated along with an urgent heart surgery with a good evolution. This case showcases the need to look for systematically systemic embolism in endocarditis of the right ventricle (RV) associated with a communication of the right and left cavities, and the possibility of an eventual paradoxical embolism within VSD in case of an inverted right to left shunt. Furthermore, it highlights that congenital VSD can be the underlying condition of a severe endocarditis in case of acute staphylococcus bacteremia.

## Introduction

The incidence of infective endocarditis in adults with a small unoperated VSD is high. Some studies report an incidence varying between 1.45 and 8/100 years [1]. The vegetations are typically located in the right heart, involving valves and most often embolize in pulmonary circulation [2]. Rarely, paradoxical embolisation in systemic circulation can occur [3]. We report a case of right-sided IE without the involvement of cardiac valves and complicated with pseudoaneurysms on the aortic arch and systemic emboli in a 27-year-old female with prior perimembranous restrictive VSD.

## Patient and observation

**Patient information:** a 27-year-old woman, with a history of documented congenital VSD, was presented to our unit with prolonged fever, chills, and weight loss for two months. Due to her poor socio-economic background, the patient neglected to do regular medical check-ups. No complications due to her congenital disease were objected to. She had a history of drug substance abuse, but no skin lesion of IV injections was found. She also had no history of a recent infection or recent dental procedure.

**Clinical findings:** on admission, she had hyperthermia (38.7°C), tachycardia (117 bpm), and normal blood pressure (100/51 mmHg). She had a respiratory rate of 24 bpm and an oxygen saturation of 98%. She was conscious but was very pale and weak.

**Figure 1:**
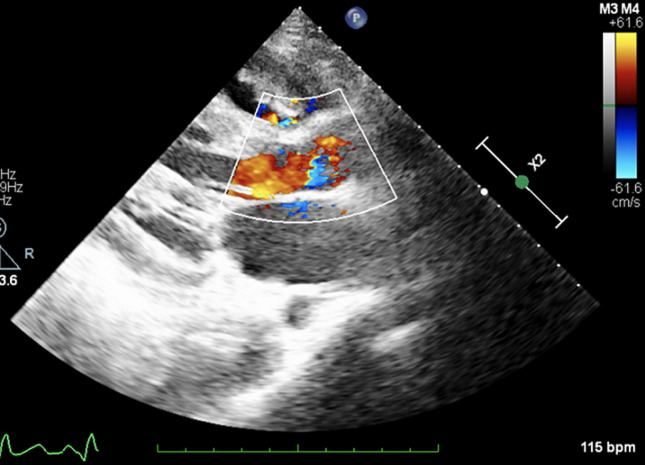
echocardiography showing the vegetation attached to the VSD

**Diagnostic assessment:** on physical examination, we noted a palpable thrill and a grade 5/6 high-pitched holosystolic murmur, along the left sternal border. No evidence of an infectious portal of entry was objected to. The electrocardiogram showed a regular sinus rhythm of 110 bpm, and revealed a right bundle branch block (RBBB) with abnormal repolarization. Her chest X-ray was normal. Her laboratory blood results found a profound anaemia (Hb = 8 g/dL), with elevated white blood cells (25000 elements/mm_3_), and an inflammatory profile (CRP = 165 mg/L, ferritin = 648 µg/L, fibrinogen = 5,10 g/L). The renal and hepatic markers were in the normal range. The search for an immunosuppressive condition such as HIV or diabetes was negative. A transthoracic echocardiography revealed a perimembranous restrictive left to right shunt VSD, with aneurysmal tissue surrounding it. Two mobile vegetations were found attached to the defect on his right side, and one was attached to the anterior face of the RV. The size of the biggest vegetation was measured (25 mm). No valvular involvement was found, and there was no pulmonary hypertension. The ventricles had normal sizes with normal functions, and left ventricular hypertrophy was objected (Figure 1). Transoesophageal echocardiography confirmed the findings.

**Figure 2:**
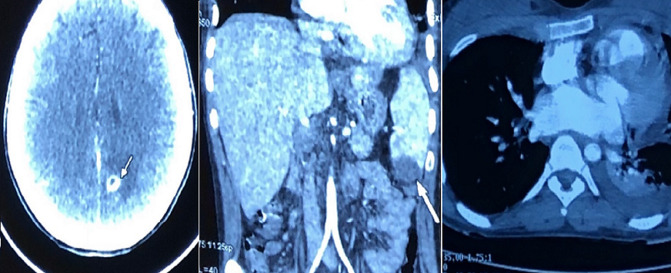
systemic emboli (cerebral, pulmonary and splenic)

**Figure 3:**
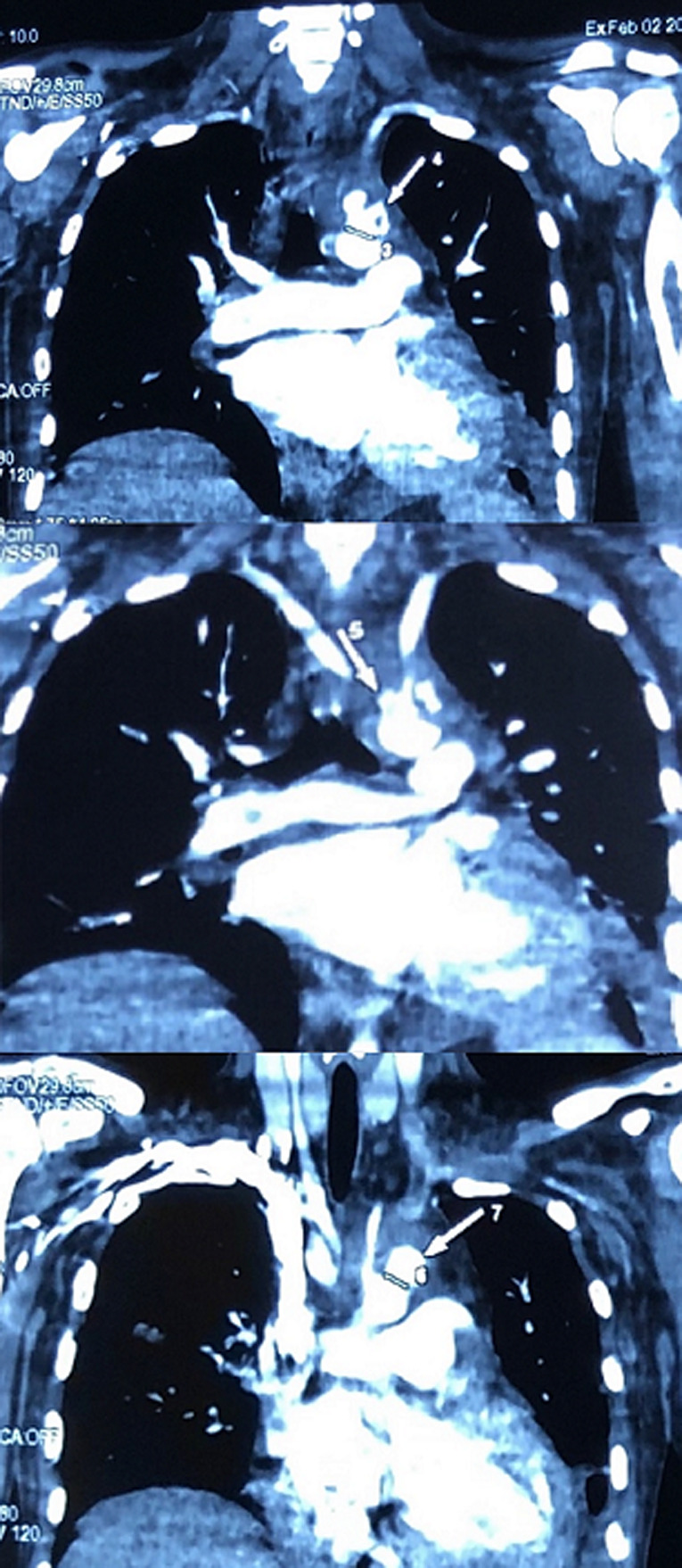
aneurysms on the cross of the aorta

The diagnosis of isolated right-sided infective endocarditis was then suggested, and further exams to complete Dukes’s criteria. Three sets of blood cultures grew Staphylococcus hominis. She was treated at first with intravenous antibiotics (third-generation cephalosporin and gentamicin), then the treatment was adapted according to the results of the antibiogram with a switch to a first-generation cephalosporin with a total duration of treatment of six weeks. A complete body CT scan showed multiple complications, such as multiple emboli in the brain, lungs, and spleen, and five mycotic aneurysms localized on the region of the aortic arch with signs of cracking with a little blood effusion in the mediastinum. An aspect of supravalvular stenosis of the ascending aorta was suspected using reconstructing imaging techniques. Interestingly, the patient had an anatomic variation concerning the supra-aortic trunks (SATs) with the right sub-clavicular artery born directly from the aorta. Nevertheless, no pulmonary arteriovenous malformation was found (Figure 2, Figure 3) The evolution was marked by persistent fever, along with persistently abnormal inflammatory biomarkers, despite an oriented antibiotic therapy.

**Figure 4:**
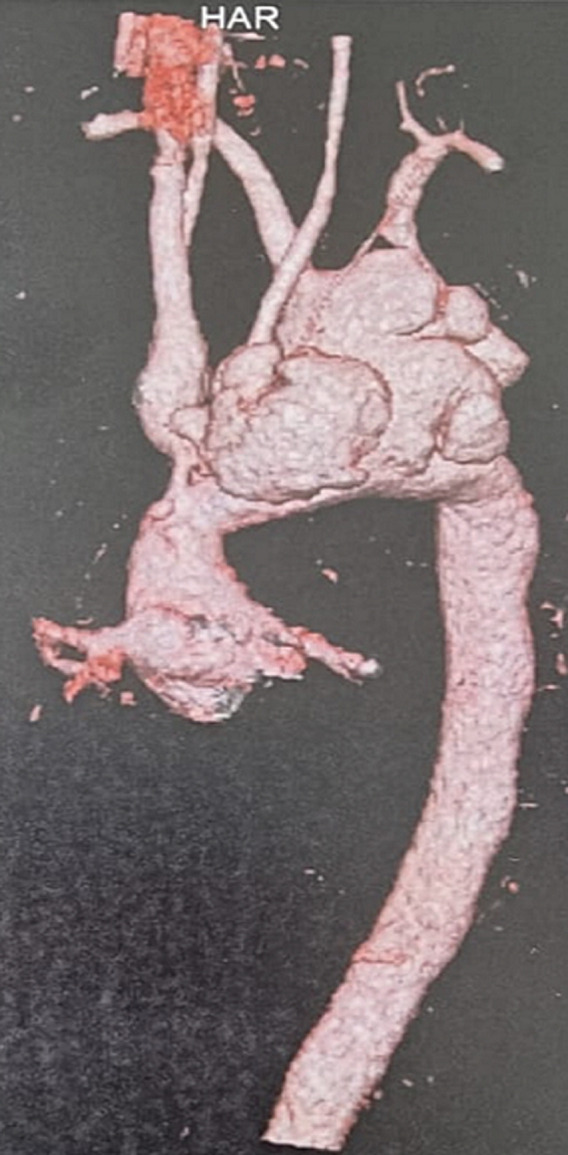
surgical view of the aortic aneurysms

**Figure 5:**
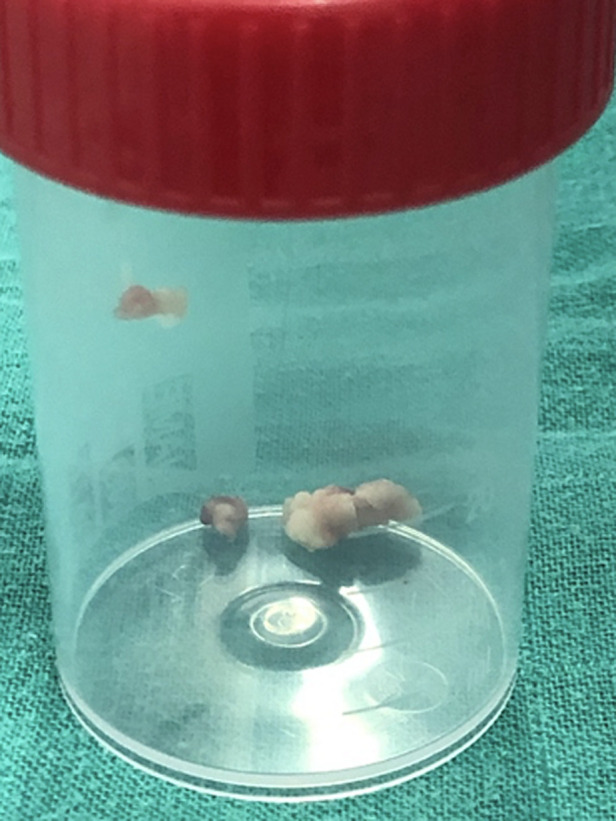
vegetations found during the surgery

**Therapeutic interventions:** considering the embolization, the size of the vegetation and sepsis evolution, an emergency surgery was indicated. The VSD was closed using sutures, after median sternotomy during cardiopulmonary bypass (CPB). Small vegetations were found in the right ventricle and were taken off. The ascending aorta was small, and the aortic arch was very dilated with enormous aneurysms. Given the difficulty to access and place the aortic clamp and the cardiogenic cannula, and the high risk of prolonged hypothermia on the brain to insert the aortic prosthesis, a later endovascular approach was preferred. After the CPB was terminated, the patient was closed up and transferred immediately to the intensive care unit (Figure 4, Figure 5).

**Follow-up and outcome of interventions:** the postoperative period was uneventful, and the patient was discharged a few weeks later after terminating the rest of the antibiotic therapy. The culture of the vegetation was negative. The short- and medium-term evolution was favorable with the decrease of the temperature curve and the inflammatory markers, as well as the improvement of the general condition of the patient.

**Informed consent:** it was obtained from the patient.

## Discussion

In congenital diseases, VSD is a condition that does not necessarily require surgery, especially if the shunt is small and not of hemodynamic importance [4,5]. Its natural history in adults can be marked by many complications, including pulmonary arterial hypertension and endocarditis, particularly if there isn´t an adequate follow-up. In the situation of infective endocarditis (IE), vegetations are often located in the right heart where the shunt is headed and involve the defect borders, the free walls of the right ventricle and the tricuspid and/or pulmonary valves. In our case, we describe an isolated right-sided infective endocarditis with no cardiac valve involvement.

Embolization when it occurs is pulmonary. Systemic emboli may be found during investigations, especially during the first week of antibiotherapy or in the event of an uncontrolled sepsis [6]. This should motivate the clinician to seek pulmonary arterial hypertension or vegetation in the left cavities, explaining the systemic complications. If none of these circumstances are found, paradoxical embolism could be evoked [7]. If present, an associate patent foramen ovale which is the most often cause of paradoxical embolism, should be searched for. In our case report, no vegetation was found in the left cavities. The first hypothesis is that vegetation was indeed present in the left ventricle or the mycotic aorta and embolized rapidly in the aorta. The second hypothesis is ultimately a paradoxical embolism, and therefore the absence of another arteriovenous malformation notably a persistent foramen ovale, suggests that the VSD is likely the only possible passage for the vegetations.

Multiple embolisms have been found at the cerebral and splenic levels. In the case of paradoxical embolism, the systemic location may be elucidated by a possible inversion of the left-to-right shunt. Classically, it is related to pulmonary etiology. In our case, we can evoke the possibility of Valsalva maneuvers including urination, defecation, and sneezing [8], as the transthoracic echocardiography has found normal pulmonary pressures. Blood cultures were positive for staphylococcus coohni, which is a species of coagulase-negative staphylococci (CoNS), normally found on the skin as part of the commensal flora. It rarely causes native valve endocarditis, and is isolated in 4.3% to 5.08% of CoNS bacteremia cases [9]. The hemoculture findings suggest skin contamination, more likely due to the patient´s drug abuse history.

Another rare trait in our case is the unusual localization of the aneurysm in the aortic arch region. It can be explained by the turbulent flow succeeding the narrow portion of the aorta, which fragilizes the aorta wall. Nevertheless, the possibility of a pre-existing aneurysm infected secondarily by the bacteremia is also plausible given the multiple malformations; but no prior scans were done to confirm and tests for autoimmune diseases were negative. To our knowledge, no similar case associating VSD complicated by right ventricle endocarditis, possible paradoxical emboli, and enormous aortic pseudoaneurysm was found.

## Conclusion

This report proves the need to systematically search for systemic emboli within VSD complicated by right heart endocarditis and all circumstances that increase intrathoracic pressure should be avoided.
